# Dual-mode endogenous and exogenous sensitization of tumor radiotherapy through antifouling dendrimer-entrapped gold nanoparticles

**DOI:** 10.7150/thno.54930

**Published:** 2021-01-01

**Authors:** Chao Yang, Yue Gao, Yu Fan, Liu Cao, Jin Li, Yulong Ge, Wenzhi Tu, Yong Liu, Xueyan Cao, Xiangyang Shi

**Affiliations:** 1 State Key Laboratory for Modification of Chemical Fiber and Polymer Materials, College of Chemistry, Chemical Engineering and Biotechnology, Donghua University, Shanghai 201620, People's Republic of China.; 2 Department of Radiation Oncology, Shanghai General Hospital, Shanghai Jiao Tong University School of Medicine, Shanghai 201620, People's Republic of China.

**Keywords:** dendrimers, gold nanoparticles, HIF-1α siRNA, gene silencing, radiotherapy

## Abstract

Development of a powerful sensitization system to alleviate radioresistance for enhanced tumor radiotherapy (RT) remains to be explored. Herein, we present a unique dual-mode endogenous and exogenous nanosensitizer based on dendrimer-entrapped gold nanoparticles (Au DENPs) to realize enhanced tumor RT.

**Methods:** Generation 5 poly(amidoamine) dendrimers partially modified with 1,3-propanesultone were used for templated synthesis of Au NPs, and the created zwitterionic Au DENPs were adopted for serum-enhanced delivery of siRNA to lead to the knockdown of hypoxia-inducible factor-1α (HIF-1α) protein and downstream genes to relieve tumor invasion. The Au DENPs/siRNA polyplexes were also used for dual-mode endogenous and exogenous sensitization of tumor RT *in vivo*.

**Results:** Due to the dual-mode endogenous sensitization through HIF-1α gene silencing and the exogenous sensitization through the existing Au component, enhanced RT of cancer cells *in vitro* and a tumor model *in vivo* can be realized, which was confirmed by enhanced cytotoxic reactive oxygen species (ROS) generation *in vitro* and double-strand DNA damage verified from the γ-H_2_AX protein expression in tumor cells *in vivo*. By integrating the advantages of HIF-1α gene silencing-induced downregulation of downstream genes and the dual-mode sensitization-enhanced RT, simultaneous inhibition of primary tumors and metastasis can be readily realized.

**Conclusions:** The developed zwitterionic Au DENPs may be used as a promising platform for dual-mode endogenously and exogenously sensitized RT of other tumor types.

## Introduction

As a common and essential method in cancer treatments, radiation therapy (RT) utilizes high-intensity ionizing radiation to inhibit tumor proliferation through cytotoxic reactive oxygen species (ROS) generation 1, 2. However, the efficiency of RT is not always satisfactory due to the fact that oxygen depletion caused by malignant proliferation can produce hypoxic areas at tumor sites, thus weakening the sensitivity of RT and causing radioresistance 3-5. The major underling mechanism is the high-level expression of hypoxia-inducible factor-1α (HIF-1α) at the tumor site, a key component of HIF-1 protein. As a key regulator, HIF-1α mediates tumor proliferation 6, angiogenesis 7, tumor invasion and metastasis 8, inducing RT resistance 9. Therefore, in order to effectively improve the therapeutic effect and maximize tumor attenuation, it is particularly necessary to develop an ideal RT sensitization system to address the RT resistance bottleneck.

With the advances in cancer nanomedicine, several kinds of radiosensitizers have been developed, such as quantum dots 10, non-metal-based nanoparticles (NPs) 11, metal-based NPs 12, 13, and so on. In particular, gold (Au) NP-based materials that have reinforced photoelectric effects and Compton effects to realize amplification of electron emmision and production of hydroxyl radicals, organic radicals and peroxidation 14, 15, have been emerging as a promising exogenous radiosensitizer 16 to induce ROS generation and double-strand DNA damage for enhanced tumor RT and anti-proliferation 4, 17. Alternatively, another strategy to knock down the HIF-1α protein expression in tumor cells has been proven to be effective to endogenously sensitize the RT for improved tumor therapy 3, 18. To lead to downregulation of HIF-1α, tumor cells are usually treated with inhibitors or genetically engineered through effective gene delivery 19, 20. However, chemical inhibitors such as YC-1 have shown many side effects in clinical trials due to their nonspecificity 19, 21. In contrast, gene silencing though HIF-1α siRNA (si-HIF-1α) delivery has been proven to be effective and less toxic 22. However, free siRNA can be easily cleared and enzymatically degraded in the process of metabolization 23. In addition, free siRNA is difficult to penetrate the cell membrane 24 due to its negatively charged nature. Therefore, a safe and effective gene delivery vector is particularly important for siRNA delivery to achieve its functionality *in vivo*.

The current gene delivery systems can be simply divided into viral and non-viral delivery. Since viral vectors are often limited due to their inherent security problems including carcinogenicity and immunogenicity 25, non-viral vector systems such as cationic poly(ethylenimine) 26, 27, poly(beta-amino ester) 28, and poly (L-lysine) 29 polymers have received a great deal of attention. However, these polymeric vectors possess cationic charge-resulted cytotoxicity and lack versatility to incorporate other inorganic exogenous sensitizers for enhanced RT of tumors, hence judicious design of a new vector system achieving dual endogenous and exogenous sensitization remains to be a challenging task.

Dendrimers, especially poly(amidoamine) (PAMAM) dendrimers, are a family of branched cationic macromolecules with good monodispersibity and well-defined architecture including a core, branched interior, and periphery functional groups 30. In our earlier work, we demonstrate that dendrimer-entrapped gold nanoparticles (Au DENPs) can be used as an excellent gene vector simultaneously solving the high amine density-resulted cytotoxicity and low gene transfection efficiency 31, 32. This is due to the fact that the entrapped Au core particles within dendrimers help preserve the 3-dimensional conformation of PAMAM dendrimers for improved gene compaction, and likewise the entrapped Au cores require to be stabilized by a portion of dendrimer terminal amine to relieve the high density amine-induced cytotoxicity 33. Previously, we have also shown that Au DENPs can be partially modified with acetamide 34, polyethylene glycol (PEG) 35, 36, β-cyclodextrin 31, 37 or zwitterion carboxybetaine acrylamide 38 for improved gene delivery. In particular, Au DENPs partially modified with zwitterions not only possess a better biocompatibility than those partially functionalized with PEG, but also show a serum-enhanced gene delivery effect due to the rendered antifouling property of vectors after zwitterionic surface modification 38, 39. Further, our previous work has proven that Au DENPs modified with nitroimidazole enable hypoxia-targeted sensitized tumor RT thanks to reinforced photoelectric effects and Compton effects of Au cores 30. However, the developed Au DENPs have not been used for gene delivery-mediated endogenous RT sensitization.

**Scheme 1:**
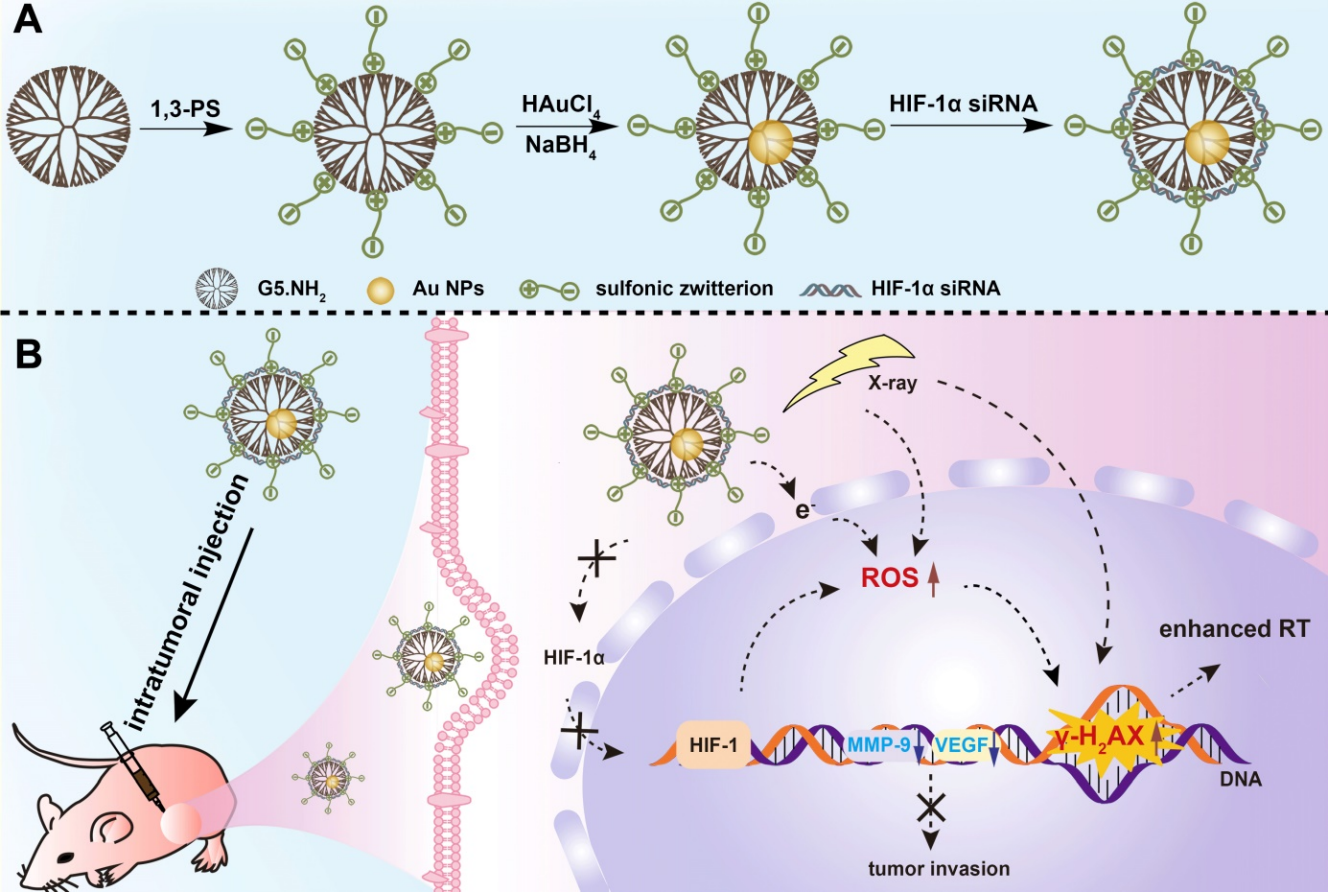
A) Schematic illustration of the preparation of {(Au^0^)_25_-G5.NH_2_-PS_20_}/siRNA polyplexes. B) The mechanism of {(Au^0^)_25_-G5.NH_2_-PS_20_}/siRNA polyplexes for combined endogenous and exogenous sensitization of tumor RT *via* HIF-1α gene knockdown and Au NPs, respectively.

In this present investigation, we aimed to develop an updated nanosystem for enhanced RT of tumors through dual-mode endogenous and exogenous sensitization based on Au DENPs (Scheme 1A-B). In this design, amine-terminated G5 dendrimers were partially decorated with 1,3-propanesultone (1,3-PS) to be rendered with antifouling property, and then entrapped with Au NPs. We systematically characterized the Au DENP vector system in terms of the structure, morphology, composition, stability, cytotoxicity, gene compaction and silencing ability, and *in vitro* dual sensitization-induced anticancer efficacy. Finally, the nanosystem was adopted for RT of a xenografted tumor model to further explore the dual sensitization effect. To the best of our knowledge, this is the first report uniquely combining both endogenous and exogenous sensitization using dendrimer nanotechnology to boost tumor RT.

## Results and Discussion

### Synthesis and characterization of Vector and Vector/siRNA polyplexes

In this work, we employed G5.NH_2_ dendrimers as a platform to generate antifouling Au DENPs as a nonviral vector. G5.NH_2_ dendrimers were first partially decorated with 1,3-PS to create zwitterionic G5.NH_2_-PS_20_ dendrimers according to the literature 38. Then, the formed G5.NH_2_-PS_20_ dendrimers were used for templated synthesis of Au NPs through a fast sodium borohydride reduction chemistry to form {(Au^0^)_25_-G5.NH_2_-PS_20_} DENPs (Scheme 1A).

**Figure 1:**
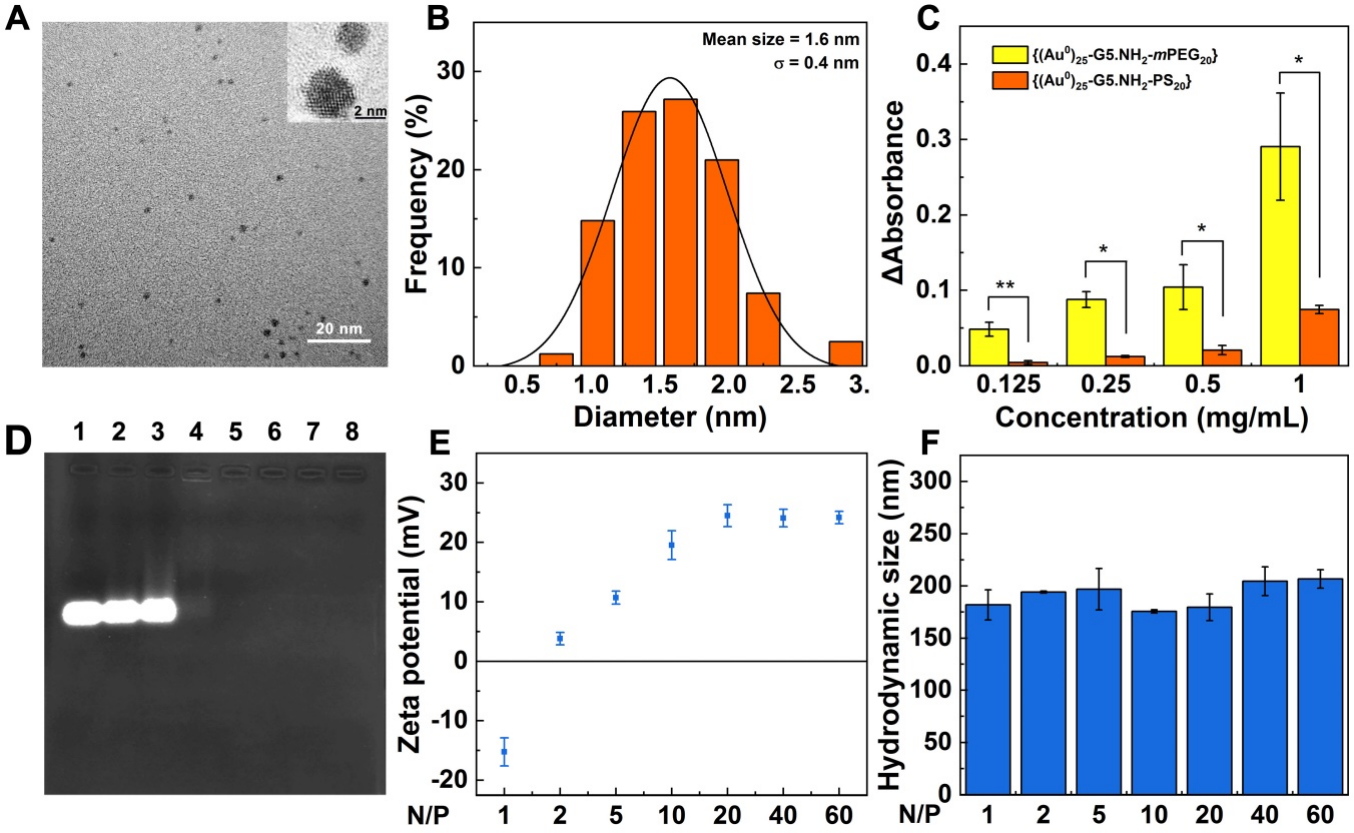
Characterization of Vector and Vector/si-HIF-1α polyplexes. **A)** TEM image and **B)** size distribution histogram of the Au core particles within the Au DENPs. Inset of (A) shows high-resolution TEM image of the Au crystals. **C)** Absorbance change of BSA/Au DENPs mixtures at 278 nm before and after incubation with {(Au^0^)_25_-G5.NH_2_-*m*PEG_20_} or {(Au^0^)_25_-G5.NH_2_-PS_20_} for 4 h, followed by centrifugation. **D)** Gel retardation assay of Vector/siRNA polyplexes under different N/P ratios. Lane 1, free si-HIF-1α; lane 2-8, N/P = 0.25, 0.5, 1, 2, 3, 4, and 5, respectively. E) Zeta potential and F) hydrodynamic size of Vector/si-HIF-1α polyplexes formed under different N/P ratios.

^1^H NMR was used to characterize the successful modification of 1,3-PS onto the dendrimer surface (Figure S1a). The peaks at 2.2-3.4 ppm represent the dendrimer methylene protons, while the peak at 1.93 ppm can be attributed to the methylene protons of the sulfonic zwitterions, indicating the successful modification of 1,3-PS. By comparison of related NMR peak integration, the number of sulfonic zwitterion moieties attached to each G5 dendrimer was estimated to be 20.4. The formation of Au DENPs was first validated by UV-vis spectroscopy (Figure S1b), where a typical shoulder band at around 520 nm can be assigned to the surface plasmon resonance band of Au NPs, in agreement with the literature 38. The size and morphology of the formed Au core particles were visualized by transmission electron microscopy (TEM, Figure 1A-B). Apparently, the Au core NPs show a spherical or semi-spherical shape with an average size of 1.6 nm. High resolution TEM (Figure 1A, inset) clearly reveals the lattice structure of Au crystals, in agreement with the literature 30. It is interesting to note that we selected the Au salt/dendrimer molar ratio at 25:1 in our study due to the fact that at this stoichiometry, the created Au DENPs exhibit the optimal gene delivery efficiency 32. Through inductively coupled plasma-optical emission spectroscopy (ICP-OES) analysis, the practical Au atom/dendrimer molar ratio for the formed Au DENPs was calculated to be 25.2: 1, quite close to the initial molar feeding ratio. The generated Au DENPs were proven to have good colloidal stability through monitoring their hydrodynamic size at room temperature within a period of at least one week (Figure S2). Obviously, the Au DENPs can keep their hydrodynamic size at about 427.4 nm without any appreciable changes during one week. It should be noted that the measured hydrodynamic size of the Au DENPs is much larger than that measured by TEM. This should be due to the different measurement mechanisms and objectives, in consistence with the literature 38.

To check if the zwitterionic modification of Au DENPs renders them with good antifouling property, protein resistance assay was carried out (Figure 1C). Bovine serum albumin (BSA) possessing a characteristic UV absorption peak at 278 nm was used as the model protein. The antifouling property of Au DENPs was quantified by measuring absorbance change of BSA/Au DENPs mixture (Δabsorbance) before and after mixing with the Au DENPs for 4 h, followed by centrifugation. For comparison, partially PEGylated Au DENPs ({(Au^0^)_25_-G5.NH_2_-*m*PEG_20_}, m represents methoxy) with the same dendrimer surface modification degree were synthesized according to our previous work 40, characterized by ^1^H NMR (Figure S3), and also tested (Figure 1C). The Δabsorbance in the {(Au^0^)_25_-G5.NH_2_-PS_20_} group is significantly smaller than that in the {(Au^0^)_25_-G5.NH_2_-*m*PEG_20_} group under all studied concentrations (*p* < 0.05), implying that the {(Au^0^)_25_-G5.NH_2_-PS_20_} DENPs have a better antifouling property than the {(Au^0^)_25_-G5.NH_2_-*m*PEG_20_} DENPs, in agreement with the literature 39.

We next prepared the {(Au^0^)_25_-G5.NH_2_-PS_20_}/si-HIF-1α polyplexes (Scheme 1A) and checked the si-HIF-1α condensation property of the vector through gel retardation assay (Figure 1D). With the increase of the N/P value, the migration of si-HIF-1α gradually decreases. At the N/P ratio of 2 (Lane 5) or above, the migration of si-HIF-1α can be fully blocked. The surface potentials of Vector/si-HIF-1α polyplexes under different N/P ratios were examined (Figure 1E). At the N/P ratio of 1, the Vector/si-HIF-1α polyplexes are negatively charged. With the increase of the N/P ratio (2 or above), the surface potential of the polyplexes gradually increases and levels off at an N/P ratio of 20 (+ 24.5 mV). This further proves that at the N/P ratio of 2 or above, si-HIF-1α can be completely compacted, in accordance with the gel retardation assay data. Dynamic light scattering (DLS) was used to detect the hydrodynamic size of the Vector/si-HIF-1α polyplexes (Figure 1F). At the N/P ratio of 2-60, the hydrodynamic diameters of the polyplexes are in the range of 175.7-206.7 nm, further indicating the good siRNA compaction ability of the vector. It is interesting to note that the hydrodynamic diameters of the polyplexes are smaller than that of the Au DENPs (427.4 nm) without siRNA complexed. This may be due to the fact that the aggregation state of Au DENPs and Au DENPs/si-HIF-1α polyplexes in aqueous solution could be very different. Likely, the Au DENPs have a greater tendency to be aggregated in an aqueous solution than the Au DENPs/si-HIF-1α polyplexes, thus resulting in a greater hydrodynamic size.

### Cytotoxicity and cellular uptake assays

**Figure 2:**
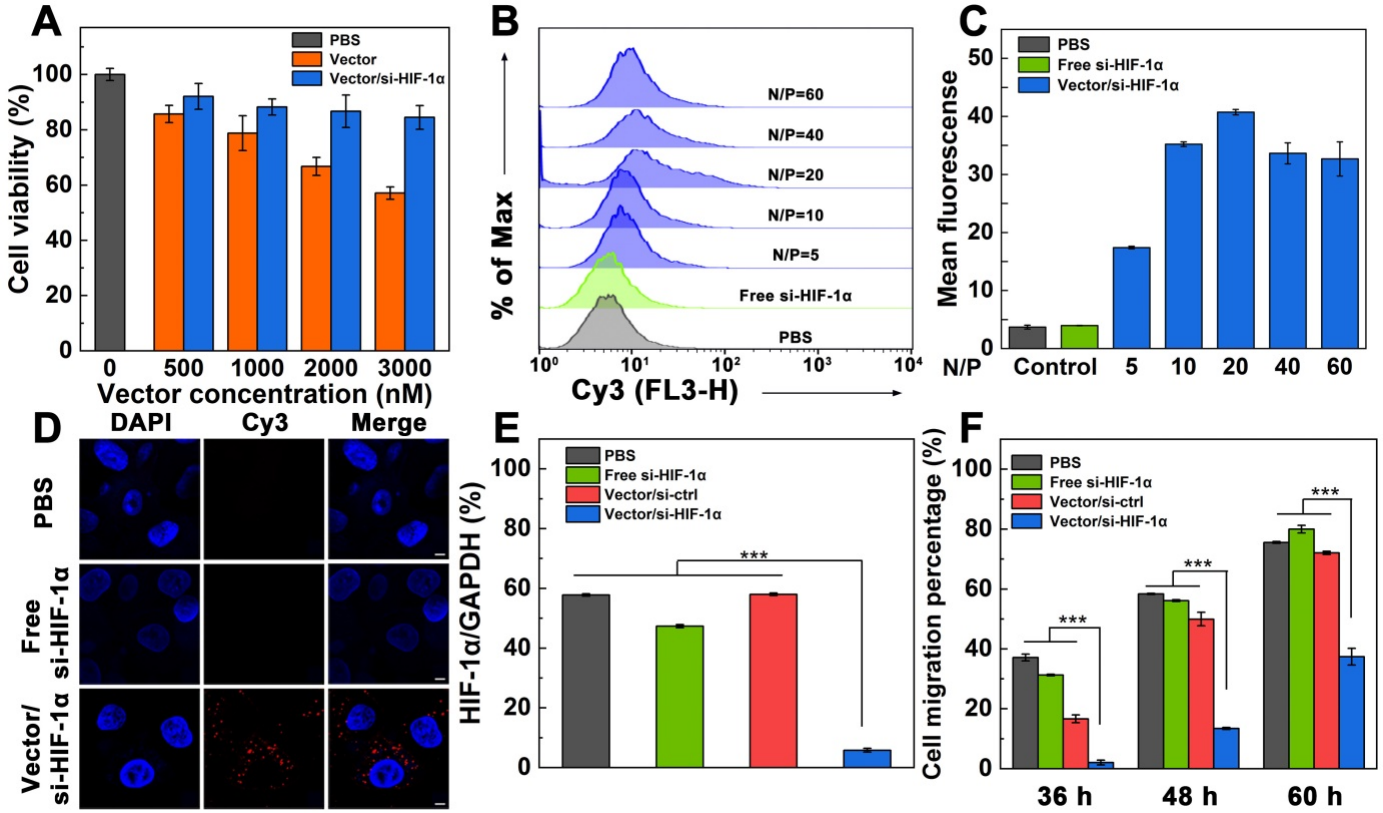
**A)** Cytotoxicity of A549 cells after treatment with Vector or Vector/si-HIF-1α under various vector concentrations. **B, C)** Flow cytometric analysis of cellular uptake of Vector/si-HIF-1α polyplexes at different N/P ratios (N/P = 5, 10, 20, 40, or 60, respectively). **D)** Confocal microscopic imaging of A549 cells treated with PBS, free si-HIF-1α, and Vector/si-HIF-1α polyplexes at an N/P of 20. Scale bar represent 5 μm for all panels. **E)** HIF-1α protein expression in A549 cells *in vitro* after different treatments. **F)** Wound-healing assay of A549 cells after treated with PBS, free-si-HIF-1α, Vector/si-ctrl polyplexes, or Vector/si-HIF-1α polyplexes at different time periods.

Cell counting kit-8 (CCK-8) assay was used to check the cytotoxicity of the Vector and Vector/si-HIF-1α polyplexes (Figure 2A). With the increase of vector concentration, the viability of A549 cells (a human non-small cell lung carcinoma cell line) gradually decreases. At the vector concentration of 3000 nM, the cell viability is less than 60%. In contrast, under the same vector concentrations tested, the viability of cells treated with the Vector/si-HIF-1α polyplexes is higher than that treated with the Vector. In particular, the cell viability remains above 85% at the vector concentration of 3000 nM. The less cytotoxicity of the polyplexes than the Vector under the same concentrations should be due to the charge neutralization of the positively charged vector after complexed with negatively charged si-HIF-1α, in consistence with the literature 38.

For effective gene transfection, it is vital to investigate the cellular uptake ability of the polyplexes. A549 cells were incubated with the polyplexes for 3 h, and analyzed through flow cytometry (Figure 2B-C). With the increase of N/P ratio, the fluorescence intensity of cells gradually increases. At the N/P ratio of 20, the mean fluorescence intensity of cells reaches the peak value (40.74), indicating that polyplexes at this N/P ratio may have the best gene transfection efficiency. To check if the zwitterionic modification of the vector enables serum-enhanced gene delivery, we separately tested the endocytosis of the si-HIF-1α in the presence or absence of serum after complexed with the vector (Figure S4). Under all the same N/P ratios, the mean fluorescence intensity of cells transfected in the presence of fetal bovine serum (FBS) is significantly higher than that in the absence of FBS (*p* < 0.05). This confirmed the utility of the zwitterionic vector for serum-enhanced gene delivery, in good agreement with our earlier work 38.

To further prove the intracellular uptake and localization of the polyplexes, A549 cells were incubated with the Vector/si-HIF-1α polyplexes at the optimized N/P ratio of 20 for 3 h, and were observed by confocal microscopy (Figure 2D). Cells treated with free Cy3-si-HIF-1α and PBS just display DAPI-stained blue cell nuclei. In sharp contrast, cells treated with the polyplexes exhibit both the DAPI-stained blue cell nuclei and the Cy3-related red fluorescence signals in the cytosol, which are associated with the internalized polyplexes. This indicates that the vector is able to successfully mediate the transmembrane delivery of si-HIF-1α into the cytoplasm.

### Knockdown efficiency of si-HIF-1α for inhibition of cell invasion

In order to evaluate the knockdown effect of the Vector/si-HIF-1α polyplexes in A549 cells, GAPDH was used as an internal control for the Western blot assay of HIF-1α protein expression (Figure 2E and Figure S5). A scramble siRNA (si-ctrl) with the same base pair to si-HIF-1α was also adopted to form Vector/si-ctrl polyplexes and transfected under the same conditions. Compared with the cells treated with PBS, free si-HIF-1α, and Vector/si-ctrl polyplexes that do not display any appreciable knockdown of HIF-1α protein, the HIF-1α protein expression in cells treated with the Vector/si-HIF-1α polyplexes is significantly down regulated (*p* < 0.001). The HIF-1α gene silencing efficiency was calculated to be 89.9% relative to the PBS control. This implies that the Vector/si-HIF-1α polyplexes enable effective knockdown of the HIF-1α protein expression.

We next checked whether the transfection of si-HIF-1α was able to inhibit the migration and metastasis of the A549 cancer cells *in vitro* through wound-healing assay (Figure S6 and Figure 2F). It can be seen that cells transfected with PBS, free si-HIF-1α, and Vector/si-ctrl polyplexes and cultured for 36 h have quite different migration rates with the cell migration percentage being 37.1%, 31.3%, and 16.7%, respectively. In contrast, cells treated with Vector/si-HIF-1α polyplexes have a very limited migration percentage (2.1%). With extension of cell incubation time period up to 48 and 60 h, the groups of PBS, free si-HIF-1α, and Vector/si-ctrl polyplexes show increased cell migration, much higher than the group of Vector/si-HIF-1α polyplexes (*p* < 0.001). In particular, after 60 h incubation, the groups of PBS, free si-HIF-1α and Vector/si-ctrl show the cell migration percentages of 75.6%, 80.0% and 72.1%, respectively, while the group of Vector/si-HIF-1α polyplexes exhibit a cell migration percentage of just 35.7%. This clearly demonstrates that cells transfected with the Vector/si-HIF-1α polyplexes display an effective anti-metastatic effect.

### Dual sensitization-boosted RT of cancer cells *in vitro*

**Figure 3:**
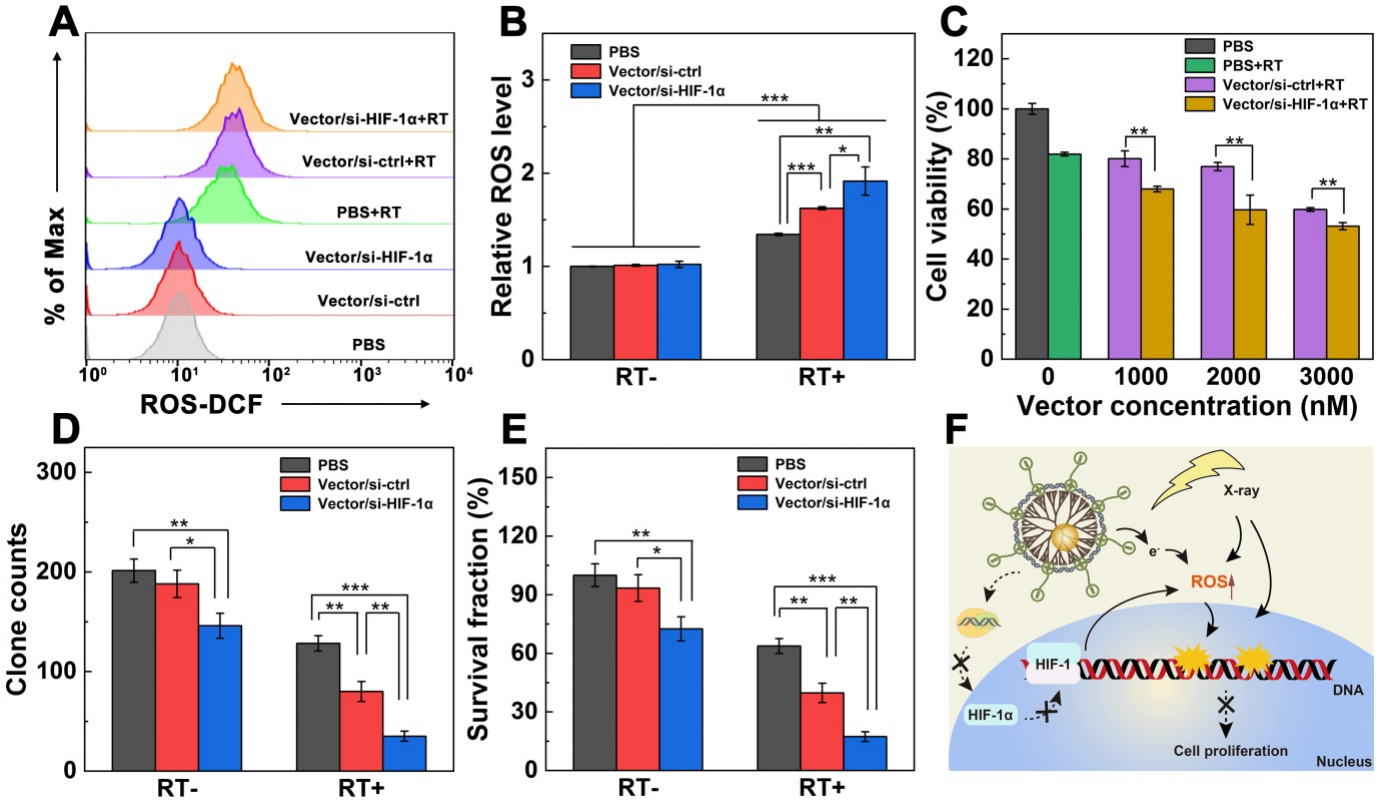
** Dual sensitization-boosted RT of A549 cells *in vitro*. A, B)** ROS generation levels of A549 cells incubated with PBS, Vector/si-ctrl, or Vector/si-HIF-1α analyzed by flow cytometry in the presence or absence of RT, respectively (N/P = 20 for both polyplexes, and radio dose of 6 Gy). **C)** Viability of cells treated with PBS, PBS+RT, Vector/si-ctrl+RT, and Vector/si-HIF-1α+RT under different dendrimer concentrations (6 Gy for RT groups), respectively. **D)** Quantification of A549 cell clones and **E)** the corresponding surviving fraction after different treatments (N/P = 20 for both polyplexes, n = 3). **F)** Proposed mechanism underlying the enhanced RT of cancer cells treated with the Vector/si-HIF-1α polyplexes.

To explore the dual sensitization-induced RT of cancer cells, we first checked the ROS generation level within cells using a fluorescence probe 2′,7′-dichlorodihydrofluorescein diacetate (DCFH-DA) through flow cytometry (Figure 3A-B). ROS level in cells treated with PBS was set to be 1.0 according to the literature 30. Compared with non-RT groups, enhanced ROS levels are found in all the RT-treated groups (*p* < 0.001). The relative ROS levels in cells follows the order of PBS+RT group (1.3) < Vector/si-ctrl+RT (1.6) < Vector/si-HIF-1α+RT (1.9). Clearly, under the same RT condition, dual sensitization through the HIF-1α gene silencing and Au NPs using the Vector/si-HIF-1α polyplexes leads to significantly higher ROS level than single RT (*p* < 0.01) and single Au NP-induced sensitization effect (*p* < 0.05).

Next, the proliferation of A549 cells incubated with Vector/si-ctrl or Vector/si-HIF-1α polyplexes under RT (6 Gy) was investigated through CCK-8 assay of cell viability (Figure 3C). With the increase of dendrimer concentration, the viability of cells treated with the Vector/si-ctrl+RT and Vector/si-HIF-1α+RT gradually decreases. At the tested concentrations (1000-3000 nM), cells treated with Vector/si-HIF-1α+RT display significantly lower viability than those treated with Vector/si-ctrl+RT (*p* < 0.01). This suggests that the Vector/si-HIF-1α polyplexes enable dual endogenous (HIF-1α gene silencing) and exogenous (Au component) sensitization of RT to improve the effect in cancer cell suppression.

To further prove the dual sensitization-induced long-term anti-proliferation effect of cancer cells, a plate clone formation test was carried out (Figure S7). Cells treated with PBS, Vector/si-ctrl, and Vector/si-HIF-1α without X-ray irradiation display apparent dense clones stained by crystal violet. As opposed, cells under the same treatment in the presence of RT display much less clones than in the absence of RT. In particular, the inhibition of clone formation follows the order of Vector/si-HIF-1α > Vector/si-ctrl > PBS. The quantitative clone counts and survival percentages data (Figure 3D-E) reveal that in the absence of RT, the HIF-1α gene silencing allows better clone inhibition than the control cells treated with PBS (*p* < 0.01) and cells treated with Vector/si-ctrl polyplexes (*p* < 0.05). Strikingly, compared with the PBS+RT group (128 clones, survival score 72.7%), the clones and survival scores of the Vector/si-ctrl+RT group (80 clones, survival score 45.3%) and the Vector/si-HIF-1α+RT group (32 clones, survival score 19.8%) significantly reduced (*p* < 0.01). Further, the dual sensitization effect in the Vector/si-HIF-1α+RT group is much more significant than the single sensitization effect in the Vector/si-ctrl group (*p* < 0.01). This again verified that both the vector and si-HIF-1α gene silencing could sensitize the RT to inhibit tumor cell proliferation.

Furthermore, the possible mechanism of dual sensitization-boosted RT of cancer cells is proposed (Figure 3F). Typically, Au NP component with a high atomic number within the vector helps inhibit cancer cell proliferation by directly inducing ROS generation to damage the double-stranded DNA 30. Likewise, the vector-enabled HIF-1α gene silencing also promotes the ROS generation. Hence, under dual sensitization, significant cancer cell suppression can be realized through RT.

### Enhanced tumor RT *in vivo*

**Figure 4:**
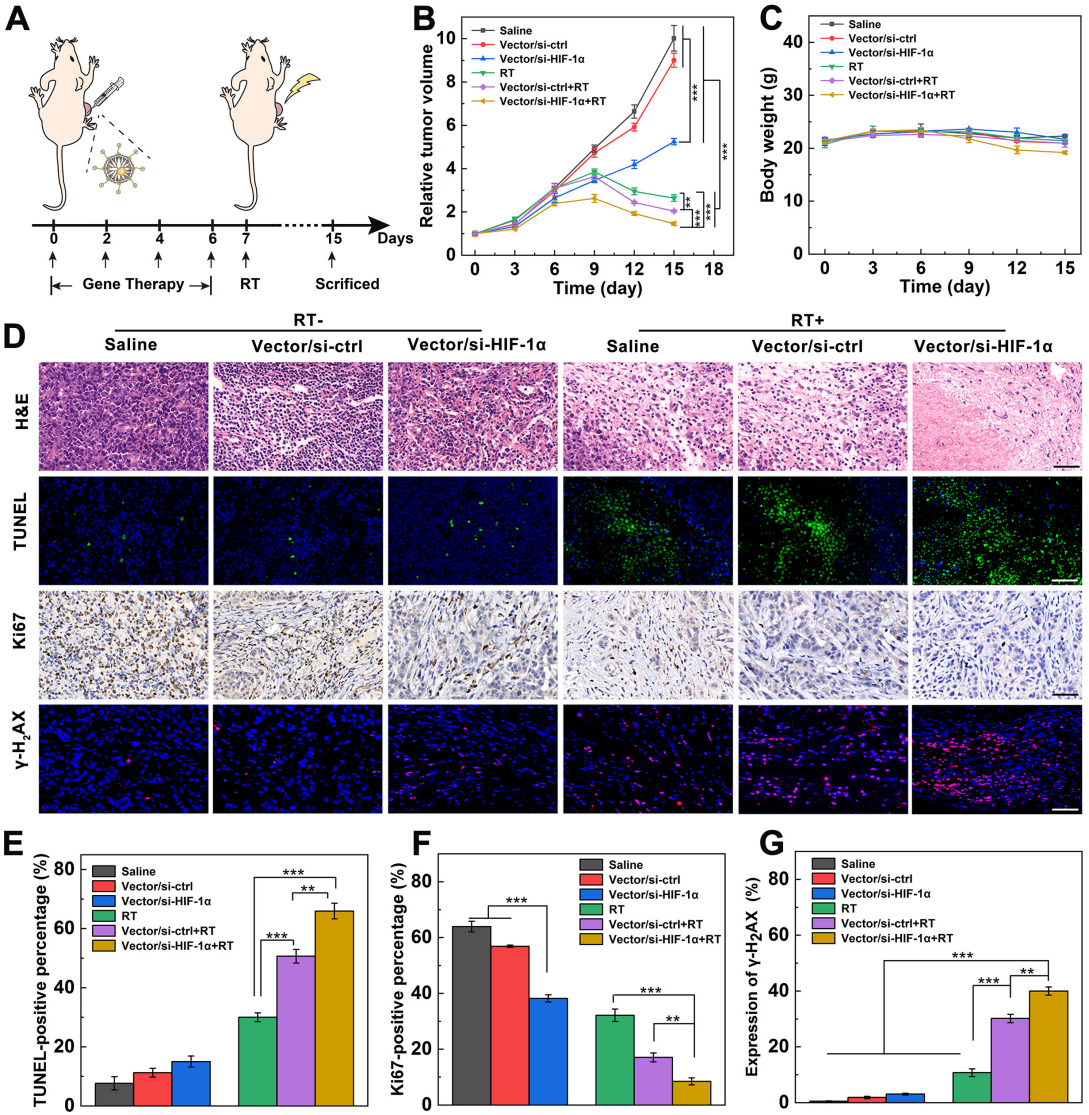
** Dual sensitization-boosted RT of tumors using Vector/si-HIF-1α polyplexes *in vivo*. A)** The process of dual sensitization using Vector/si-HIF-1α polyplexes for tumor RT. **B)** Relative tumor growth curves and **C)** body weight of mice after different treatments. **D)** Immunohistochemical and immunofluorescence images and **E-G)** quantitative analysis of TUNEL-, Ki67-, and γ-H_2_AX-stained tumor slices (scale bar = 50 μm for each panel in D), respectively.

To determine the tumor growth inhibition by Vector/si-HIF-1α+RT *in vivo*, mice bearing A549 tumors were randomly divided into six groups including saline, Vector/si-ctrl and Vector/si-HIF-1α with or without RT (6 Gy), respectively. The treatment schedule of Vector/si-HIF-1α+RT is shown in Figure 4A. It should be noted that we chose to perform RT on the seventh day because at this time point, the *in vivo* gene delivery treatment was already repeated for 4 times and the last tumor treatment lasted for 24 h. This ensured that the HIF-1α gene silencing began to be effective. As shown in Figure 4B, compared to the groups of saline and Vector/si-ctrl without RT that show negligible tumor suppression effect, the tumors treated with Vector/si-HIF-1α without RT display much smaller relative volume at 15 days post treatment (5.3, *p* < 0.001). This implies that the HIF-1α gene silencing actually avails the suppression in tumor growth. In the presence of X-ray irradiation, all three groups have much better tumor inhibition effect than the corresponding one without X-ray (*p* < 0.001). In particular, the Vector/si-ctrl and Vector/si-HIF-1α groups exhibit much higher tumor inhibition effect after 15 days than the single RT group (*p* < 0.01) due to the sensitization role played by Au NPs for Vector/si-ctrl and by both Au NPs and si-HIF-1α silencing for the Vector/si-HIF-1α, respectively. The smallest tumors are found in the Vector/si-HIF-1α+RT group (Figure S8 and Figure S9), indicating the best treatment efficacy of Vector/si-HIF-1α+RT among all groups. Likewise, all treatment groups with or without RT do not seem to induce any appreciable changes in the body weight of mice, manifesting preliminarily the biosafety of Vector/siRNA polyplexes and the selected RT dose (Figure 4C). Furthermore, biodistribution (Figure S10) and H&E staining (Figure S11) also confirmed the biosafety of the Vector/si-HIF-1α polyplexes. Due to the intratumoral injection, the Au content is mainly located at the tumor site, and only negligible Au distribution can be seen in other major organs such as heart, liver, spleen, lung and kidney at different time points. Gradual accumulation of Au can be found in the kidney, showing that the polyplexes may be able to be cleared through kidney at 48 h (Figure S10). Likewise, as shown in Figure S11, no appreciable organ damage and abnormality is observed in the H&E-stained sections of the heart, liver, spleen, and kidney organs for all treatment groups.

To further prove the dual sensitization-mediated antitumor effect, tumor tissues of different groups were collected and stained (Figure 4D). H&E staining of tumor sections shows that Vector/si-HIF-1α+RT treatment induces the most significant tumor necrosis and cell nucleus decomposition among all groups. In contrast, there are only scattered necrotic tumor cells after Vector/si-ctrl+RT or RT treatment. As shown in the TUNEL-stained images (Figure 4D) and the quantitative cell apoptosis rate analysis (Figure 4E), the Vector/si-HIF-1α+RT (66.0%) induced much more apoptotic tumor cells than Vector/si-ctrl+RT (50.7%) and RT alone (30.0%) and all other treatments without RT. Further Ki67-staining images of tumors (Figure 4D) and quantitative analysis of Ki67-positive cell percentages (Figure 4F) show that the brown signals representing the Ki67-positive proliferative cells have the most significant decrease in the Vector/si-HIF-1α+RT group among all other groups. In particular, the percentages of Ki67-positive cells for the Vector/si-HIF-1α+RT group is 8.5%, much lower than those of Vector/si-ctrl+RT (17.1%) and single RT (32.2%) groups and all other non-RT groups.

Lastly, as is known, γ-H_2_AX is a sensitive and critical cellular marker reflecting the double-stranded DNA break. All three groups with RT treatments show visible red fluorescence signals associated with the γ-H_2_AX formation (Figure 4D). Further quantitative analysis of γ-H_2_AX-positive fluorescence signals (Figure 4G) reveals that the γ-H_2_AX expression follows the order of single RT (10.8%) < Vector/si-ctrl+RT (30.2%) < Vector/si-HIF-1α+RT (40.0%). Apparently, γ-H_2_AX expression in all RT groups are significantly higher than that in all non-RT groups (*p* < 0.001), and dual sensitization through both endogenous gene silencing and exogenous Au component allows for better tumor cell DNA break than single sensitization through exogenous Au component (*p* < 0.01) and single RT (*p* < 0.001). These γ-H_2_AX staining results were further validated by Western blot assay of γ-H_2_AX protein expression in tumor cells (Figure S12), verifying the dual sensitization-induced DNA break for sensitized tumor RT.

### Anti-metastasis of tumors *in vivo*

**Figure 5:**
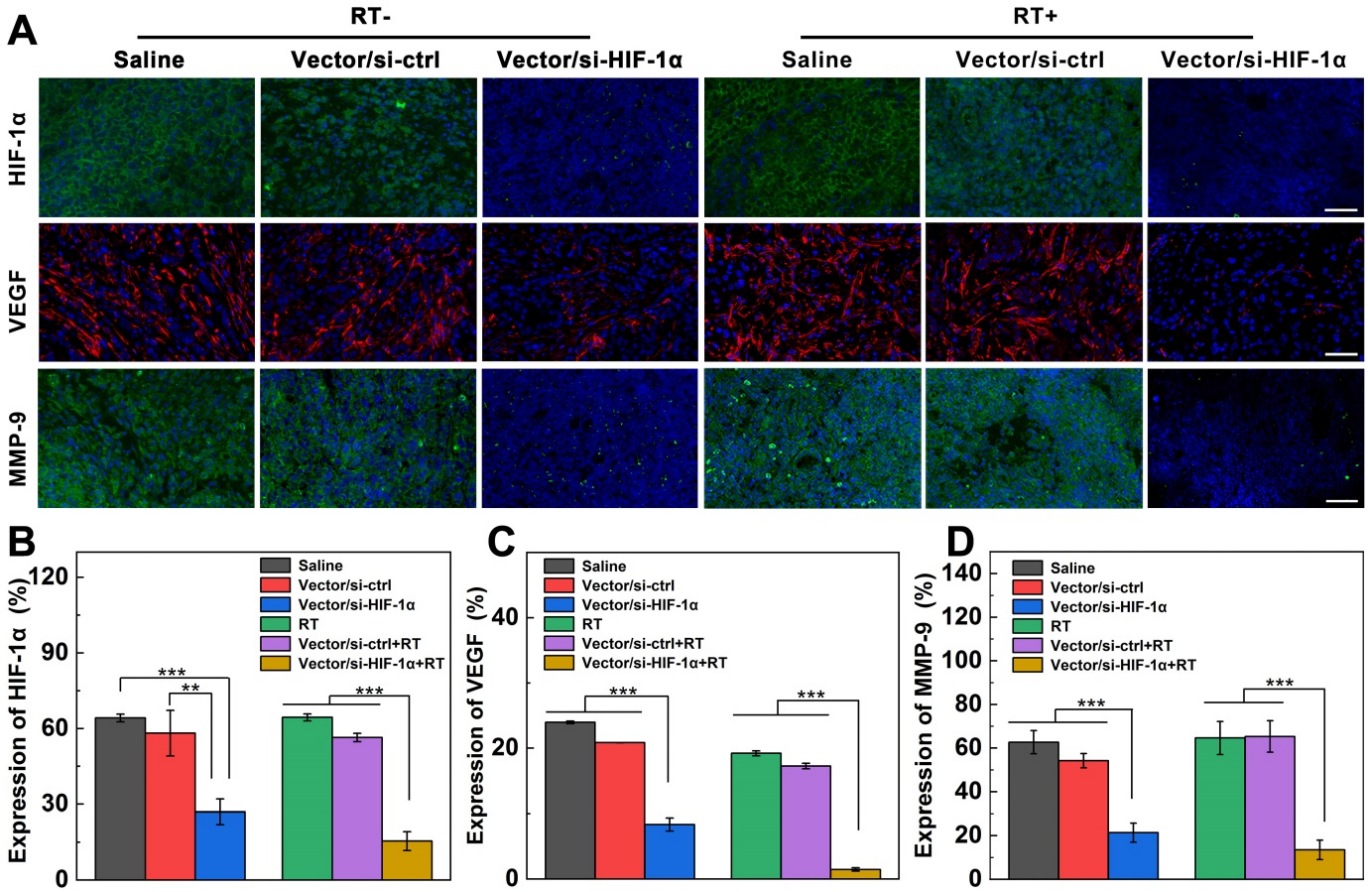
** Expression of HIF-1α and downstream invasion-related genes after different treatments. A)** Immunofluorescence images and **B-D)** quantification of tumor slices stained with HIF-1α (green), VEGF (red), and MMP-9 (green) antibodies after intratumoral injection of saline, Vector/si-HIF-1α, Vector/si-HIF-1α with or without X-ray irradiation (vector = 5 mg/kg, N/P = 20, and 100 µL saline) at 8 days post treatment according to the treatment schedule. The scale bar represents 50 µm for each panel.

HIF-1α, as a key regulation protein to induce RT resistance, also plays a pivotal role in tumor angiogenesis, invasion and metastasis 9. To prove if the successful si-HIF-1α delivery causes the inhibition of downstream signaling molecules, immunofluorescence imaging of HIF-1α, matrix metalloproteinase 9 (MMP-9), and vascular endothelial growth factor (VEGF) in tumor sections after different treatments was carried out. As shown in Figure 5A-B, the HIF-1α content (stained with green fluorescence) in the Vector/si-HIF-1α and Vector/si-HIF-1α+RT groups is significantly downregulated. The same silencing tendency was also confirmed by Western blot analysis of HIF-1α protein expression in tumor cells (Figure S13a). In addition, VEGF implicating the tumor angiogenesis and metastasis 41, 42 is also remarkably knocked down after si-HIF-1α delivery into tumor tissues by Vector/si-HIF-1α polyplexes (Figure 5A, C). The red fluorescence associated to VEGF expression in the Vector/si-HIF-1α+RT group is only about 1.5%, far less than that in both RT alone (19.2%) and Vector/si-ctrl+RT (17.3%) groups (*p* < 0.001). Western blot analysis of VEGF protein expression (Figure S13b) gave the similar supporting results, implying that down-regulation of HIF-1α causes the inhibition of the downstream VEGF protein expression closely related to tumor angiogenesis and metastasis. Moreover, MMP-9, another signal marker of tumor invasion and metastasis regulated by HIF-1α 42, is also downregulated to 21.4% in the Vector/si-HIF-1α group, much more significant than in the Vector/si-ctrl (54.2%) and saline (62.7%) groups (Figure 5A, D). The MMP-9 downregulation was further enhanced after RT treatment, where the regulation rate of Vector/si-HIF-1α group (13.5%) is much lower than those of the Vector/si-ctrl+RT (65.3%) and single RT (64.6%) groups. Western blot assay (Figure S13c) further validated the downregulation of MMP-9 protein in tumor cells after transfection of the Vector/si-HIF-1α polyplexes.

To investigate the effective anti-metastasis of tumors after dual sensitization-boosted tumor RT, H&E staining of the lung tissues was performed (Figure S14). A panoramic scanning of H&E sections shows that some areas of metastatic lesions with condensed tumor cell nuclei distinct from normal tissues are distributed in the lungs of all groups except Vector/si-HIF-1α and Vector/si-HIF-1α+RT groups. These results indicate that the developed dendrimer-based vector enables efficient siRNA transfection to silence HIF-1α gene, thus leading to downregulation of various metastasis-related genes and effective prevention of lung metastasis eventually.

## Conclusions

To conclude, we developed an updated nanomedicine formulation related to zwitterion-modified Au DENPs complexed si-HIF-1α for dual sensitization-boosted RT of tumors. We show that partial modification of 1,3-PS avails the vector of good antifouling property for serum-enhanced transfection of si-HIF-1α to knock down the HIF-1α protein expression. Owing to the presence of exogenous Au component and the endogenous knockdown of HIF-1α gene, the RT of cancer cells *in vitro* is significantly boosted as proven by increased ROS generation and decreased cancer cell invasion and clone formation. Furthermore, the dual sensitization of the Vector/si-HIF-1α polyplexes boosted effective tumor RT *in vivo*, which was confirmed by histological examinations showing the DNA damage, significant HIF-1α protein knockdown, and alleviation of tumor invasion and metastasis *via* downregulation of VEGF and MMP-9 proteins. Overall, our study represents one of the advanced designs to uniquely integrate exogenous and endogenous sensitizers to boost tumor RT through dendrimer nanotechnology, which may be extended to sensitized RT of other tumor types.

## Experimental Section

### Synthesis of {(Au^0^)_25_-G5.NH_2_-PS_20_} dendrimer-entrapped nanoparticles (DENPs)

The {(Au^0^)_25_-G5.NH_2_-PS_20_} DENPs were prepared according to the literature 43. First, G5 PAMAM dendrimers were modified with 1,3-PS by adding 20 molar equiv. of 1,3-PS (3.51 μL, 1.392 g/mL) into a G5.NH_2_ water solution (50 mg, 50 mL) under stirring at room temperature for 1 day. The formed raw product of G5.NH_2_-PS_20_ dendrimers was added with HAuCl_4_·4H_2_O (687 μL, 30 mg/mL in water) under an ice bath condition. After stirring for 15 min, the dendrimer/Au salt mixture was rapidly added with NaBH_4_ (5.67 mg, in 1 mL cold water) while stirring for 3 h to form a wine-red Au colloid dispersion. The reaction mixture was dialyzed against water (6 times, 2 L) through regenerated cellulose membranes with an molecular weight cut-off of 500 for 3 days, and then lyophilized to get the {(Au^0^)_25_-G5.NH_2_-PS_20_} DENPs.

### Preparation and characterization of DENPs/siRNA polyplexes

DENPs/siRNA polyplexes were prepared under different N/P ratios (the molar ratio of primary amines of the dendrimers to phosphates in the siRNA backbone) according to the literature 44. The DENPs with different amounts were dispersed in water and mixed with 1 or 5 μg siRNA according to different N/P ratios, respectively. The polyplexes were incubated at room temperature for 15-30 min before further characterization or transfection.

### Cell culture evaluation

A549 cells were cultured to assess the cytocompatibility of Vector or Vector/siRNA polyplexes, the cellular uptake efficacy of the polyplexes, the HIF-1α gene silencing efficiency and subsequent cell invasion inhibition. In addition, the ROS generation, cell proliferation, and clone formation were also assessed after cells were transfected with the Vector/si-HIF-1α polyplexes with or without RT.

### Sensitization of tumor RT *in vivo*

All animal experiments were conducted under the guidelines of the Animal Care and Use Committee of Donghua University, and also in accordance with the policy of the National Institute of Health. The tumor RT sensitization effects were confirmed by tumor size and body weight measurements, immunofluorescence/immunochemistry examinations of H&E, TUNEL, Ki67, γ-H_2_AX, HIF-1α, VEGF, and MMP-9 markers, and Western blotting of γ-H_2_AX, HIF-1α, VEGF and MMP-9 protein expressions in tumor cells. Biodistribution of the polyplexes and H&E staining of the major organ slices were also performed. See full experimental details in the Supplementary Information.

## Supplementary Material

Supplementary information and figures.
