# Supplementary material for: Dual-mode endogenous and exogenous sensitization of tumor radiotherapy through antifouling dendrimer-entrapped gold nanoparticles
Source: Theranostics. 2021 Jan 1;11(4):1721–31. doi: 10.7150/thno.54930 (PMC7778585; doi:10.7150/thno.54930)
Supplement: Supplementary file 1 — Supplementary information and figures. [file thnov11p1721s1.pdf]

Supporting Information

Dual-mode endogenous and exogenous sensitization of tumor radiotherapy through antifouling dendrimer-entrapped gold nanoparticles

Chao Yang[a], Yue Gao[a], Yu Fan[a], Liu Cao[a], Jin Li[a], Yulong Ge[b], Wenzhi Tu[b], Yong Liu[b], Xueyan Cao[a], and Xiangyang Shi[a]*

[a] State Key Laboratory for Modification of Chemical Fibers and Polymer Materials, College of Chemistry, Chemical Engineering and Biotechnology, Donghua University, Shanghai 201620, People's Republic of China

[b] Department of Radiation Oncology, Shanghai General Hospital, Shanghai Jiao Tong University School of Medicine, Shanghai 201620, China

Keywords: dendrimers; gold nanoparticles; HIF-1α siRNA; gene silencing; radiotherapy

---

* Corresponding author. E-mail: xshi@dhu.edu.cn

Experiment Section

Materials

Ethylenediamine core amine-terminated generation 5 poly (amidoamine) (PAMAM) dendrimers ($G5.NH_2$) were purchased from Dendritech (Midland, MI). 1,3-Propanesultone (1,3-PS) were obtained from J&K Scientific (Shanghai, China). Gold chloride ($HAuCl_4 \cdot 4H_2O$), sodium borohydride ($NaBH_4$) and all the other chemicals and solvents were obtained from Sinopharm Chemical Reagent Co., Ltd. (Shanghai, China). Water used in all experiments was purified using a PURIST UV Ultrapure Water System (RephiLe Bioscience, Ltd., Shanghai, China) with a resistivity higher than 18.2 MΩ·cm. Regenerated cellulose dialysis membranes with a molecular weight cut-off (MWCO) of 500 were acquired from Fisher (Pittsburgh, PA). 6-Diamidino-2-phenylindole (DAPI) was purchased from Bestbio Science (Nanjing, China). HIF-1α siRNA (for short, si-HIF-1α, sense: 5′-UGUGAGUUCGCAUCUUGAUTT-3′, and antisense: 5′-AUCAAGAUGCGAACUCACAUU-3′) and scramble siRNA (for short, si-ctrl, sense: 5′-UUCUCCGAACGUGUCACGUTT-3′, and antisense: 5′-ACGUGACACGUUCGGAGAATT-3′) were provided by Gene-Pharm Co., Ltd. (Shanghai, China). A549 cells (a human non-small cell lung carcinoma cell line) were obtained from Cell Bank of the Chinese Academy of Sciences (Shanghai, China). F-12K medium, penicillin, streptomycin, fetal bovine serum (FBS) and trypsin (0.25%) were from Thermo Scientific (Waltham, MA). Cell counting kit-8 (CCK-8) and 2′,7′-dichlorodihydrofluorescein diacetate (DCFH-DA) were purchased from Beyotime Biotechnology (Shanghai, China). Agarose was from Biowest (Nuaille, France). LuriaBertani (LB) medium, 4S Green plus (a safe nucleic acid dye to replace ethidium bromide), GAPDH antibody and the Tissue or Cell Total Protein Extraction Kit were from Sangon Biotech Co., Ltd. (Shanghai, China). HIF-1α antibody was from Santa Cruz Biotechnology, Inc. (Dallas, Texas). γ-H_2AX antibody was from Abconal Technology (Wuhan, China). TUNEL assay kit, VEGF antibody and MMP-9 antibody were from Servicebio (Wuhan, China).

Synthesis of {$(Au^0)_{25}$-$G5.NH_2$-PS_{20}} Dendrimer-Entrapped Nanoparticles (DENPs)

The {$(Au^0)_{25}$-$G5.NH_2$-PS_{20}} DENPs were prepared according to the literature [1]. First, G5

PAMAM dendrimers were modified with 1,3-PS by adding 20 molar equiv. of 1,3-PS (3.51 μL, 1.392 g/mL) into a G5.NH_2 water solution (50 mg, 50 mL) under stirring at room temperature for 1 day. The formed raw product of G5.NH_2-PS_{20} dendrimers was added with $HAuCl_4 \cdot 4H_2O$ (687 μL, 30 mg/mL in water) under ice bath. After stirring for 15 min, the dendrimer/Au salt mixture was rapidly added with $NaBH_4$ (5.67 mg, in 1 mL cold water) while stirring for 3 h to form wine-red Au colloid dispersion. The reaction mixture was dialyzed against water (6 times, 2 L) through regenerated cellulose membranes with an MWCO of 500 for 3 days, and then lyophilized to get the {$(Au^0)_{25}$-G5.NH_2-PS_{20}} DENPs.

Characterization Techniques

^{1}H NMR measurements were performed on a Bruker DRX 500 NMR spectrometer (400 MHz). Samples were dissolved in D_2O before measurements. UV-vis spectra were recorded using a Lambda 25 UV-vis spectrophotometer (PerkinElmer, Boston, MA). The concentration of Au for the {$(Au^0)_{25}$-G5.NH_2-PS_{20}} DENPs was determined by Leeman Prodigy inductively coupled plasma-optical emission spectroscopy (ICP-OES, Hudson, NH). The number of the primary amines of Au DENPs was determined using a Megazyme PANOPA Assay Kit (Wicklow, Ireland) according to the manufacturer's instruction. Before measurements, the Au DENPs were dispersed in water (2 mg/mL). Transmission electron microscopy (TEM) was used to observe the size and morphology of the formed Au core particles of the Au DENPs. The {$(Au^0)_{25}$-G5.NH_2-PS_{20}} DENPs were dispersed in water (2 mg/mL), deposited on a carbon-coated copper grid, and air dried before TEM measurements using a JEOL 2100F analytical electron microscope (Tokyo, Japan) at an accelerating voltage of 200 kV.

Protein resistance assay of {$(Au^0)_{25}$-G5.NH_2-PS_{20}} DENPs was carried out according to the literature [2]. UV-vis spectroscopy was employed to check the absorbance of bovine serum albumin (BSA, 1mg/mL, in PBS)/Au DENPs mixture at 278 nm at the time of mixture formation after incubation with the Au DENPs at different concentrations (0.125, 0.25, 0.5 and 1 mg/mL, respectively) at 37 ºC for 4 h. After that, the mixtures were centrifuged and the supernatant was measured. The reduced absorbance at 278 nm for the BSA/Au DENPs mixture was calculated to quantify the antifouling property of the Au DENPs.

Preparation and Characterization of DENPs/siRNA polyplexes

DENPs/siRNA polyplexes were prepared under different N/P ratios (the molar ratio of primary amines of the dendrimers to phosphates in the siRNA backbone) according to the literature [3]. The DENPs with different amounts were dispersed in water and mixed with 1 or 5 μg siRNA according to different N/P ratios, respectively. The polyplexes were incubated at room temperature for 15-30 min before further characterization or transfection.

For gel retardation assay, we prepared 1% (w/v) agarose gel containing 4S Green plus, and then according to the N/P ratios of 0.25:1, 0.5:1, 1:1, 2:1, 3:1, 4:1 and 5:1, the vector/siRNA polyplexes were prepared (1 μg siRNA/well) by co-incubation at room temperature for 15-30 min. Naked siRNA (1 μg) was also used for comparison**.** Next, the 6 × loading buffer was mixted with the vector/siRNA polyplexes. The mixtures with different N/P ratios were added into the wells and electrophoresis was performed at 80 V for 20 min. The retardation of siRNA was imaged using a ChemiDoc™ XRS+ Molecular Imager (BIO-RAD, Hercules, CA).

Surface potentials and hydrodynamic sizes of the polyplexes under various N/P ratios (1: 1, 2: 1, 5: 1, 10: 1, 20: 1, 40: 1 and 60: 1, respectively) were determined *via* a Malvern Zetasizer Nano ZS system (Worcestershire, UK) equipped with a standard 633-nm laser. To prepare the vector/siRNA poplyplexes, the vector of $\{(Au^0)_{25}\text{-G5.NH}_2\text{-PS}_{20}\}$ DENPs and 5 μg siRNA were incubated at room temperature for 15-30 min, then water was added to make the final volume of each sample reach 1 mL before measurements.

Cytotoxicity Assay

A549 cells were regularly cultured and passaged at 37 ºC in a humidified atmosphere of 95% air and 5% CO_2 using F-12K medium supplemented with 10% FBS and 1% penicillin-streptomycin. To check the cytotoxicity of vector or vector/siRNA polyplexes, A549 cells were seeded in 96-well plates at a density of 1×10^4 cells per well in 0.1 mL of F-12K medium overnight. When the degree of adherence of cells reached 60%-70%, the medium of each well was replaced with fresh medium containing the vector or vector/si-HIF-1α polyplexes with a dendrimer concentration ranging from 0 to 3000 nM (1 μg of siRNA for each polyplex). After that, the cells were continueously cutured for 24

h, added with CCK-8 agent and incubated for additional 3 h. The cell viability was measured using a CCK-8 kit according to the manufacturer's instructions. The cells in each well were analyzed using a Thermo Scientific Multiskan MK3 ELISA reader (Waltham, MA) at a wavelength of 450 nm. Each sample was tested in six parallel wells. Cells treated with PBS were used as control, and the corresponding cell viability was recorded as 100%.

Cellular Uptake of Vector/si-HIF-1α Polyplexes

A549 cells were seeded in 12-well plates at a density of 2.5×10^5 cells per well with 1 mL F-12K medium, then cultured overnight to reach 80% cell adherence. The next day, each well of cells was replaced with fresh medium (1 mL) containing vector/si-HIF-1α polyplexes (0.1 mL in PBS), which were prepared at an N/P value of 5, 10, 20, 40, or 60 (1 μg Cy3-labeled HIF-1α siRNA for each well of the culture plate). Cells treated with PBS and free HIF-1α siRNA under the same conditions were used as controls. The cells were regularly incubated for 3 h, digested with trypsin, and collected to detect the Cy3 red fluorescence intensity by flow cytometry (BD FACS Calibur, Franklin lakes, NJ). FSH-H/SSC-H was set as the cell gate and FL3-H as the Cy3 fluorescence channel.

To observe the intracellular uptake of the vector/si-HIF-1α polyplexes, A549 cells were seeded in confocal culture dishes at a density of 2×10^5 cells per dish with 1 mL F-12K medium, and cultured overnight till the adherence of cells reached 80%. Then, the medium of each dish was replaced with fresh complete F-12K medium (1 mL) containing vector/si-HIF-1α polyplexes (0.1 mL in PBS) with an optimized N/P ratio of 20 (1 μg si-HIF-1α for each dish) and the cells were incubated for 3 h. Cells treated with PBS and free HIF-1α siRNA under the same conditions were used as controls. After that, the cells were washed three times with PBS, fixed with 4% glutaraldehyde at 4 °C for 15 min, stained with DAPI for 15 min, washed with PBS for 3 times, and observed to check their red fluorescence signals with a ZEISS LSM-700 laser scanning confocal microscope (Jena, Germany).

Western Blot Assay

To analyze the si-HIF-1α transfection-induced HIF-1α protein expression inhibition, A549 cells were seeded in a 6-well plate at a density of 5×10^5 cells per well with 1 mL F-12K medium, and cultured overnight to bring the cells to 60-70% confluence. Then the medium of each well was

replaced with fresh complete F-12K medium (1 mL) containing vector/si-HIF-1α or vector/si-ctrl polyplexes (0.1 mL in PBS) at an optimized N/P ratio of 20 (1 μg siRNA per well) and the cells were incubated for 3 h. Cells treated with naked si-HIF-1α and PBS were used as control. After that, the medium of each well was exchanged with fresh complete medium and the cells were continueously incubated for 48 h. Afterwards, the cells were washed with cold PBS for three times, and lysed to extract protein with the Tissue or Cell Total Protein Extraction Kit. The extracted total protein was electrophoresed with sodium dodecyl sulfate polyacrylamide gels, followed by transferring to PVDF membranes (Millipore, Billerica, MA). The membrane was blocked in 5% non-fat milk, incubated with primary antibody at 4 °C overnight, and incubated with secondary antibody. The density of target protein signals was observed using a chemiluminescence imaging system (Merck Millipore, Burlington, MA). All antibodies were diluted in PBS, and the membrane was washed with PBS between steps. GAPDH was used as an internal control.

Inhibition of Cancer Cell Metastasis after HIF-1α siRNA Transfection

Wound healing assay was performed according to protocols described in the literature [4]. A549 cells were seeded into 12-well plates at a density of 5×10^4 cells per well with 1 mL medium and cultivated overnight. The next day, a scratch was made using a 100-μL tip in each well, and the cells were rinsed three times with PBS to get rid of the floating cells. Then, each well of cells was added with 900 μL fresh complete medium and 100 μL vector/si-HIF-1α or vector/si-ctrl polyplexes in PBS containing 1.0 μg si-HIF-1α or si-ctrl at an N/P ratio of 20. After 3 h incubation, the medium of each well was exchanged with fresh complete medium and images of the wound were taken by a Leica DM IL LED inverted phase contrast microscope after 0 h, 36 h, 48 h and 60 h, respectively (0 h represents cells before treatment). Quantitative analysis of the cell migration rate was carried out using ImageJ software (https://imagej.nih.gov/ij/download.html).

Relative Reactive Oxygen Species (ROS) Level Assay

ROS assay was performed according to the literature [5, 6]. A549 cells were seeded in 6-well plates at a density of 5×10^5 cells per well with 1 mL F-12K medium, and were cultured overnight till the adherence of cells reached 60-70%. Then, the medium of each well was replaced with fresh

complete F-12K medium (1 mL) containing vector/si-HIF-1α or vector/si-ctrl polyplexes (0.1 mL in PBS) at an N/P ratio of 20 (1 μg siRNA per well) and the cells were transfected for 3 h. Cells treated with PBS served as control. After cultivation for 24 h, the cells were irradiated with/without 6 Gy (indicated by RT+/RT-). Then, the cells were stimulated with a fluorescence probe (DCFH-DA, 1: 1000 dilution) for 1 h. Next, the cells were collected for flow cytometry assay of the green fluorescence of the probe at the FL1-H channel to check the ROS level of the cells.

Cell Proliferation Assay

A549 cells were seeded in 96-well plates at a density of 5×10^3 cells per well with 100 μL F-12K medium and cultivated overnight. Then, the medium of each well was exchanged with 100 μL fresh complete medium containing 10 μL vector/siRNA polyplexes in PBS prepared at a dendrimer concentration ranging from 0 to 3000 nM (1 μg of siRNA for each sample). After 24 h culture, cells were treated without and with 6 Gy of X-ray irradiation. Then the cells were continueously cultured for additional 48 h, and the cell viability was evaluated using a CCK-8 kit according to the manufacturer's instructions. See also details in the section of Cytotoxicity Assay.

Colony Formation Assay

A549 cells were seeded in 6-well plates at a density of 2×10^3 cells per well with 1 mL F-12K medium and cultivated overnight. Then the medium of each well was replaced with fresh complete F-12K medium (1 mL) containing vector/siRNA polyplexes (0.1 mL in PBS) at an N/P ratio of 20 (1 μg siRNA per well) to transfect the cells for 3 h. Cells treated with PBS served as blank control. After co-culture for 24 h, the cells were irradiated without or with 6 Gy X-ray irradiation. Then, cells were continuously cultured for 10-12 days until colonies became visible, stained with crystal violet solution, photographed and counted according to the literature [7].

Enhanced Radiation Therapy in A549 Bearing Mice

All animal experiments were conducted under the guidelines of the Animal Care and Use Committee of Donghua University, and also in accordance with the policy of the National Institute of Health. Five-week-old BALB/c male nude mice were obtained from Shanghai Slac Laboratory Animal Center (Shanghai, China). To build up an A549 tumor model, each mouse was subcutaneously

implanted with an A549 cell suspension (1×10^7 cells, in 100 μL PBS) in its right flank. Ten days later, when the tumor volume reached approximately 50 mm^3, the mice were divided into six groups to be treated with i) Saline; ii) Vector/si-ctrl; iii) Vector/si-HIF-1α; iv) RT; v) Vector/si-ctrl + RT; and vi) Vector/si-HIF-1α + RT, respectively. Gene therapy (Vector = 5 mg/kg, siRNA = 4 μg, in 100 μL saline for each mouse) was carried out on day 0, 2, 4 and 6, respectively. X-ray radiation therapy (6 Gy) was performed at 24 h after the last gene therapy. The tumor size and body weight were recorded every three days, and tumor volume was calculated according to the formula of $width^2 \times length/2$. Hematoxylin and eosin (H&E) staining of major organs and tumors was performed on day 15 according to the literature [6, 7].

Immunofluorescence and Immunochemistry

Tumor slices were obtained from mice sacrificed at 24 h after RT treatment (on the 8th day in the schedule shown in Figure 5a). HIF-1α antibody, γ-H_2AX antibody, TUNEL assay kit, VEGF antibody and MMP-9 antibody were used for the immunofluorescence staining analysis, while Ki67 antibody and H&E staining were used for the immunochemistry staining analysis, respectively accoding to the literature protocols [6, 7]. All staining images were collected using a Nikon Imaging System (Tokyo, Japan) and analyzed with ImageJ Software.

Western Blot Analysis *in Vivo*

In order to corroborate the results of immunofluorescence, tumor tissues were collected at 48 h for Western blot analysis after the last gene therapy treatment (on the 8th day in the schedule shown in Figure 5a). Similar to Western blot assay at the cell level, the corresponding primary antibodies of γ-H_2AX, HIF-1α, VEGF or MMP-9 were incubated with proteins in cell lysates overnight at 4 °C, then the relevant secondary antibodies were added to incubate for 50 min at 37 °C. The membranes were detected with an enhanced chemiluminescent reagent (Merck Millipore, Burlington, MA).

Statistical Analysis

One-way ANOVA statistical analysis was performed to evaluate the significance of the experimental data between groups. A p value of 0.05 was selected as the significance level, and the data were indicated with * for $p < 0.5$, ** for $p < 0.01$, and *** for $p < 0.001$, respectively.

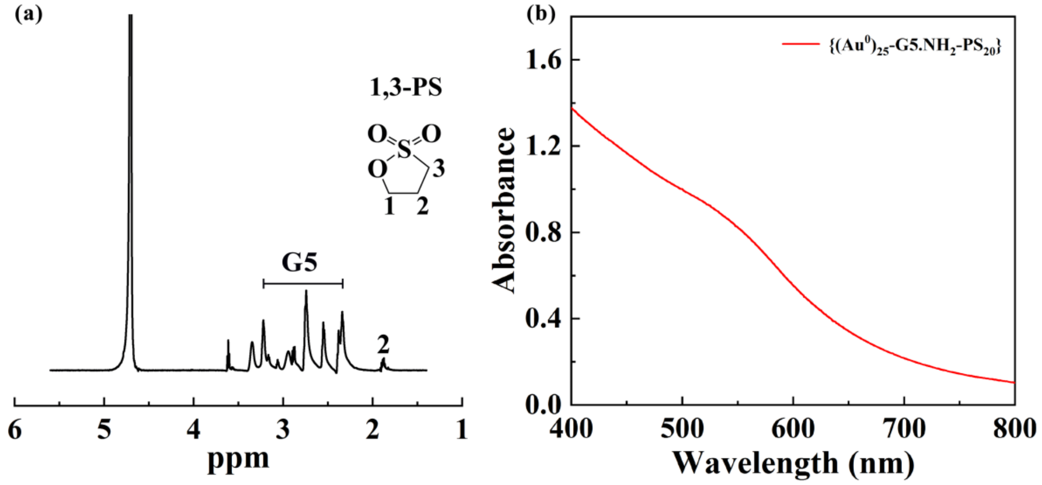

Figure S1. (a) ^{1}H NMR and (b) UV-vis spectrum of {$(Au^0)_{25}$-G5.NH_2-PS_{20}} DENPs. Sample was dispersed in D_2O or water before measurements.

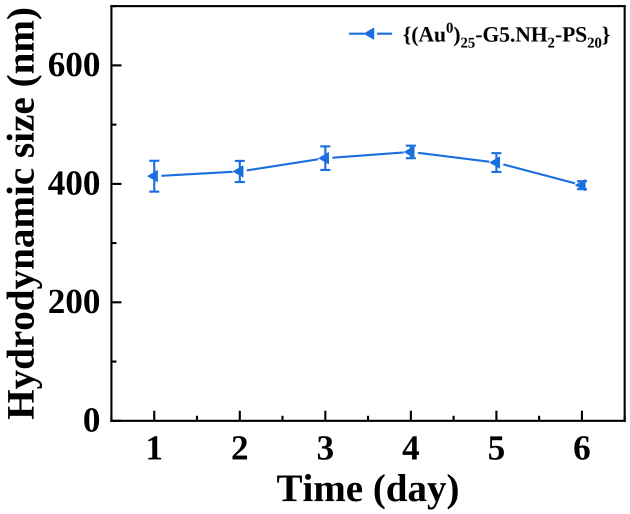

Figure S2. The hydrodynamic size of the {$(Au^0)_{25}$-G5.NH_2-PS_{20}} DENPs at room temperature at different time points.

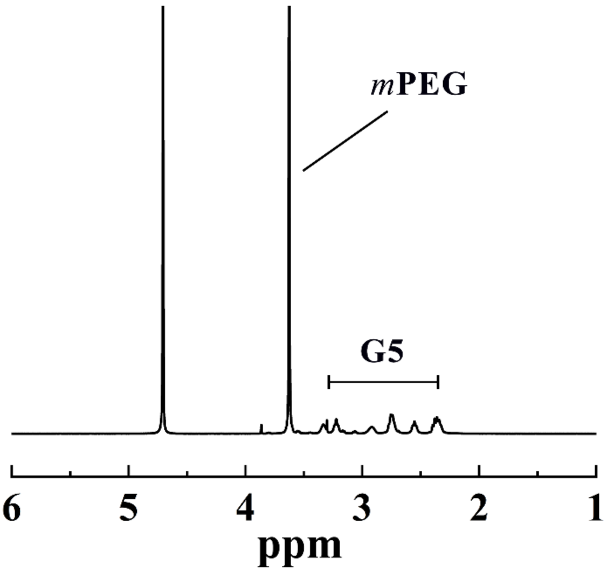

Figure S3. ^{1}H NMR spectrum of {$(Au^0)_{25}$-G5.NH_2-*m*PEG_{20}} DENPs.

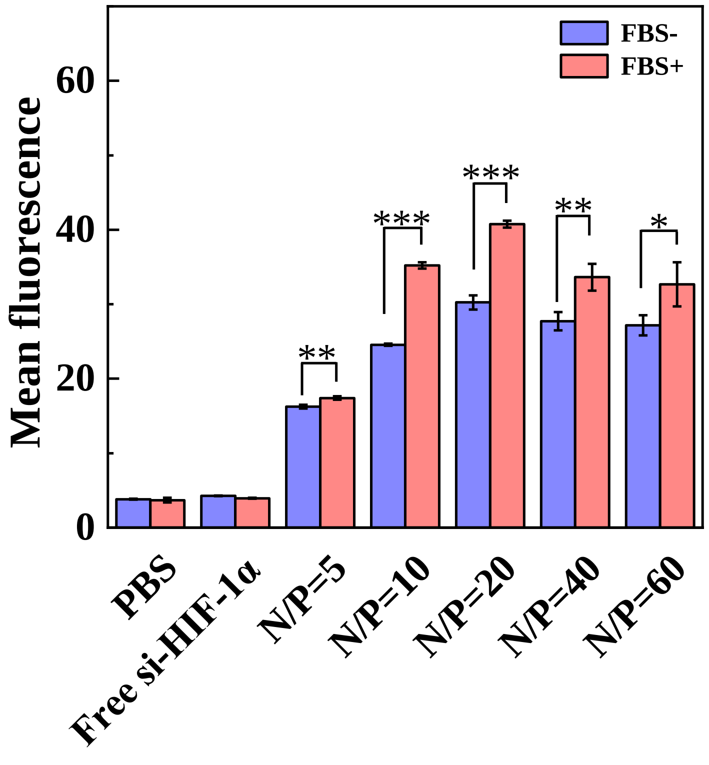

Figure S4. Flow cytometric analysis of A549 cells treated with vetcor/si-HIF-1α polyplexes at different N/P ratios in the presence or absence of FBS. Cells treated with PBS or free si-HIF-1α were used as control.

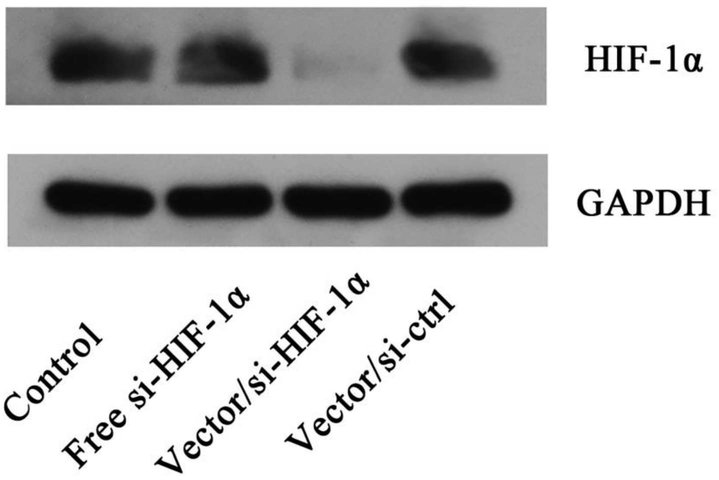

Figure S5. Western blot analysis of HIF-1α expression in A549 cells after the cells were treated with different materials for 3 h, followed by continuous culture for 48 h.

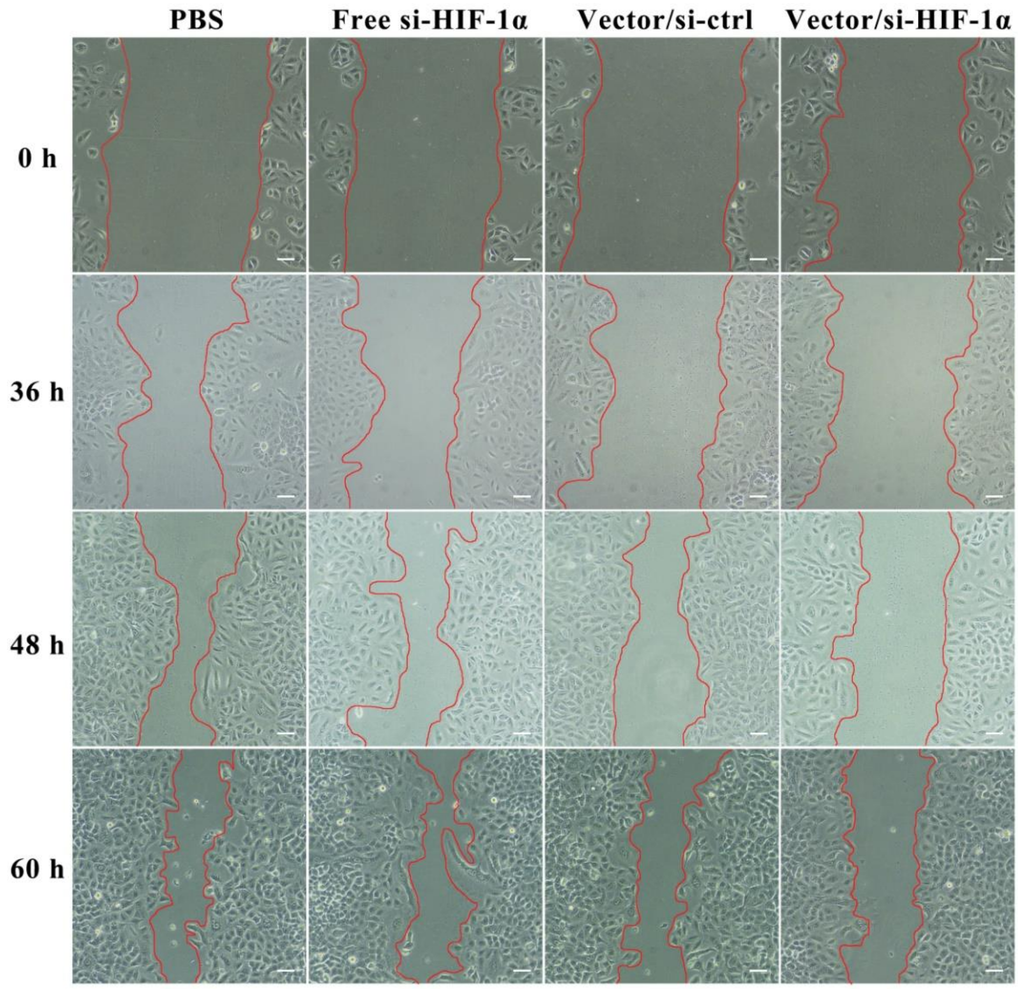

Figure S6. Wound-healing assay of A549 cells before and after transfection with PBS, free si-HIF-1α, vector/si-ctrl polyplexes, or vector/si-HIF-1α polyplexes for 3 h, followed by incubation with fresh complete medium for 36 h, 48 h and 60 h, respectively. The scar bar in each panel represents 100 μm.

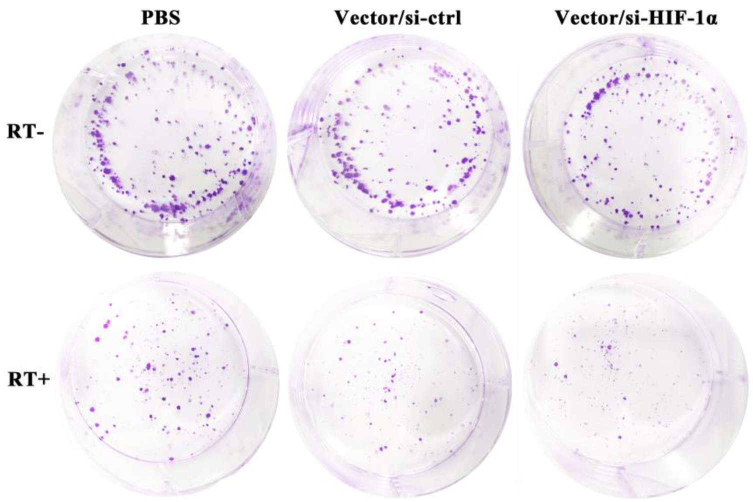

Figure S7. Colony formation images after cells were treated with PBS, vector/si-ctrl polyplexes, or vector/si-HIF-1α polyplexes in the presence or absence of X-ray irradiation (6 Gy).

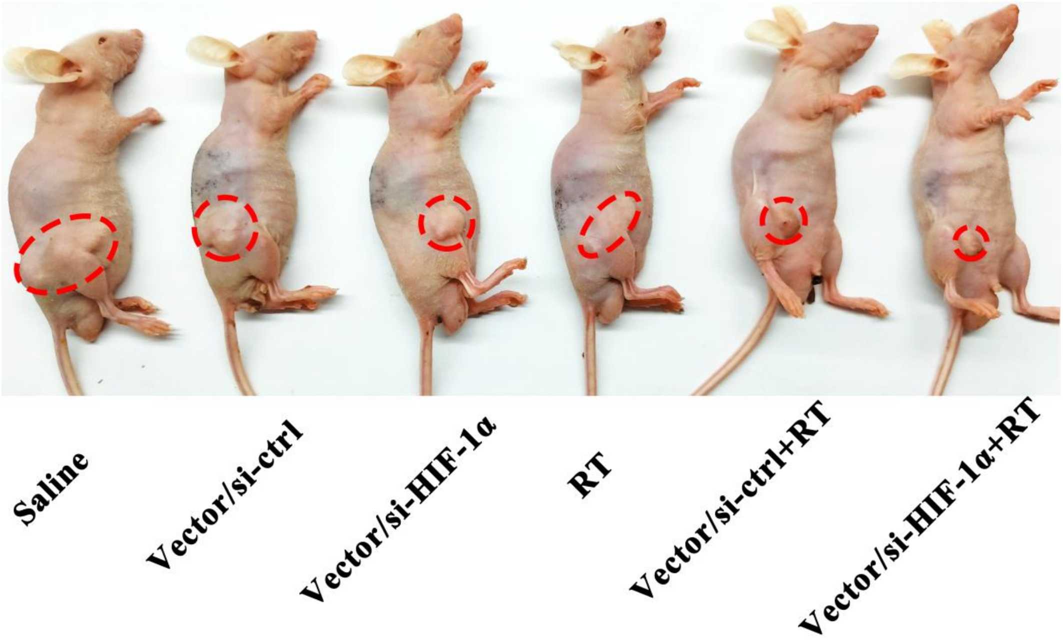

Figure S8. Representative photos of tumor-bearing mice in different groups after 15 days' treatment.

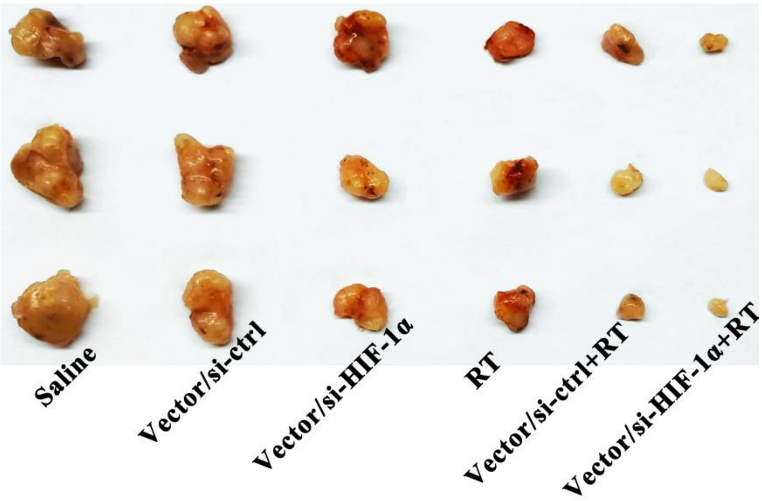

Figure S9. Photos of extracted tumors in different groups after 15 days' treatment.

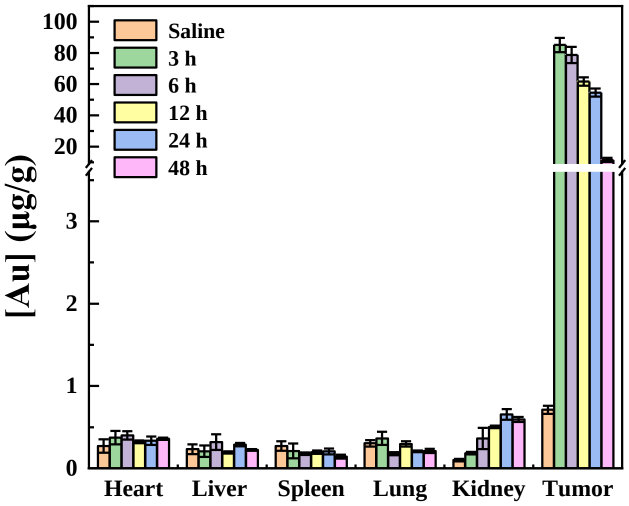

Figure S10. Biodistribution of Au element after intratumoral injection of vector/si-HIF-1α polyplexes to each tumor-bearing mouse (5 mg/kg, in 100 μL saline, n = 3).

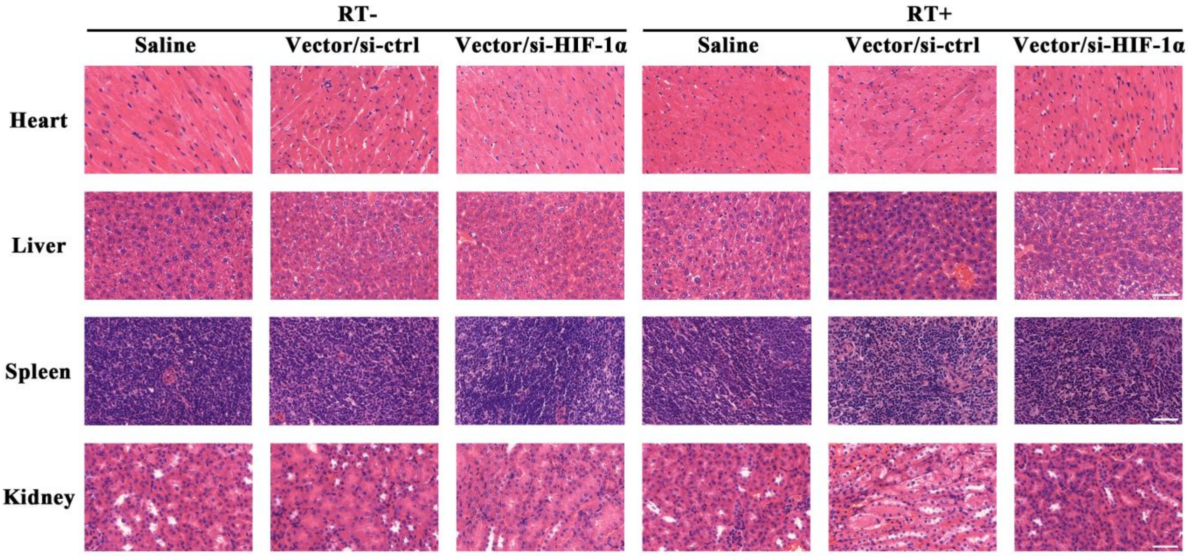

Figure S11. H&E staining of major organs of mice including heart, liver, spleen, and kidney after 15 days' treatment in different groups. Scale bar in each panel represents 50 μm.

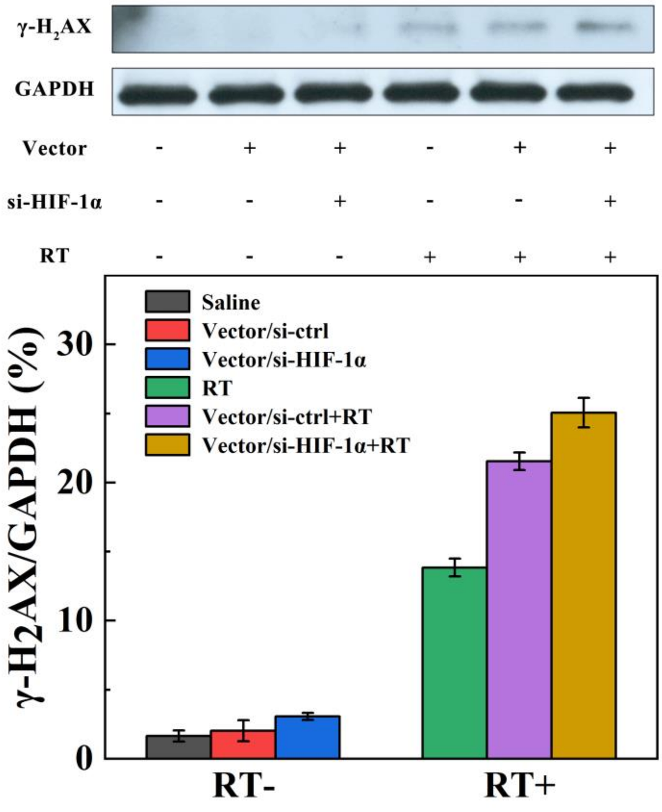

Figure S12. (a) γ-H_2AX expression levels and (b) quantitative analysis of γ-H_2AX protein expression in tumor cells after different treatments at 8 days post treatment (according to the treatment schedule) by Western blotting.

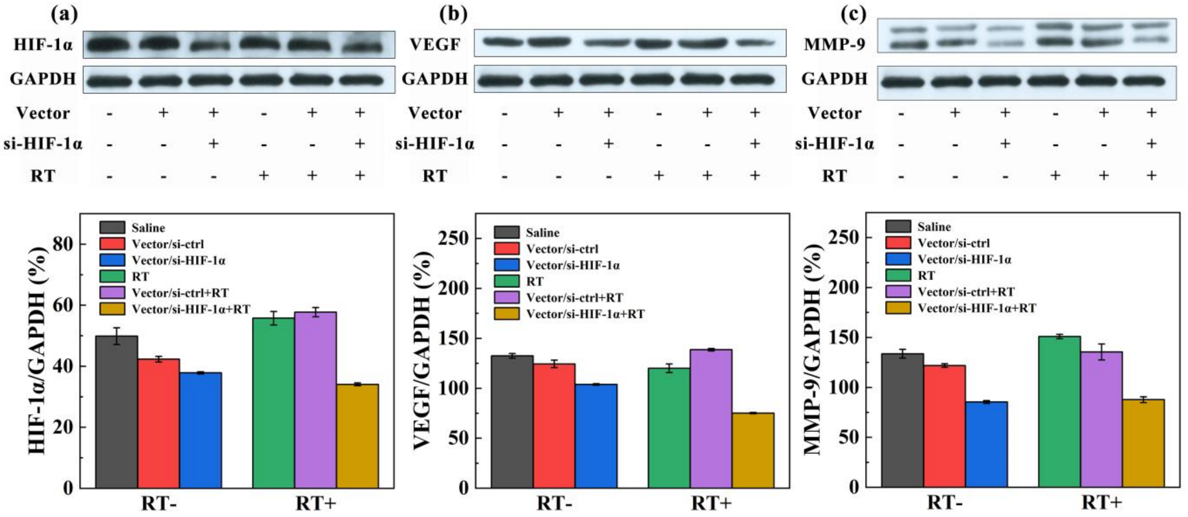

Figure S13. Western blot assay of HIF-1α protein (a) and downstream invasion-related VEGF (b) and MMP-9 (c) protein expression *in vivo*.

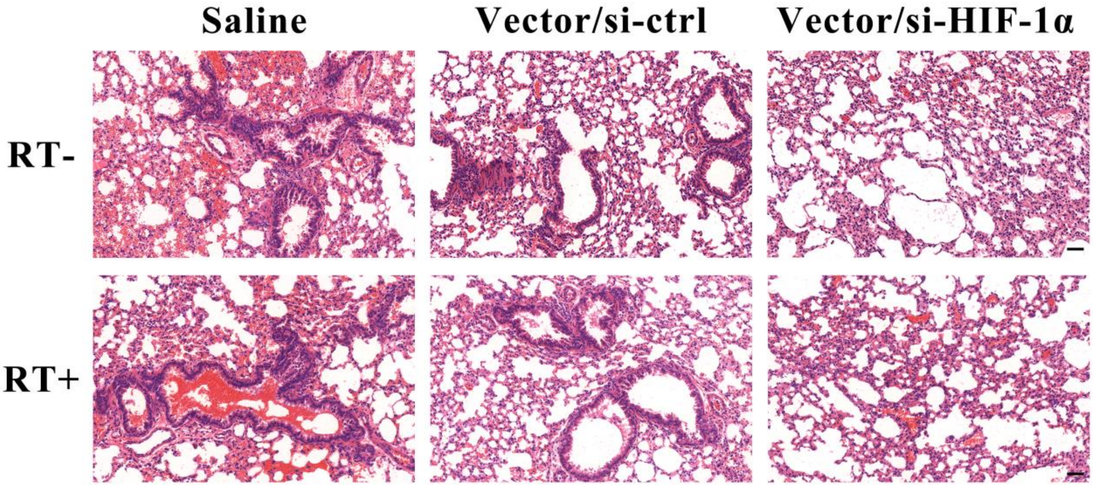

Figure S14. H&E-stained images of lung slices in different treatment groups at 15 days post treatment. Scale bar represents 50 μm for each panel.

References

1. Liu JY, Xiong ZJ, Zhang JL, Peng C, Klajnert-Maculewicz B, Shen MW, et al. Zwitterionic Gadolinium(III)-Complexed Dendrimer-Entrapped Gold Nanoparticles for Enhanced Computed Tomography/Magnetic Resonance Imaging of Lung Cancer Metastasis. ACS Appl Mater Interfaces. 2019; 11: 15212-21.

2. Xiong ZJ, Wang Y, Zhu JY, Li X, He Y, Qu J, et al. Dendrimers meet zwitterions: development of a unique antifouling nanoplatform for enhanced blood pool, lymph node and tumor CT imaging. Nanoscale. 2017; 9: 12295-301.

3. Song C, Xiao YC, Ouyang ZJ, Shen MW, Shi XY. Efficient co-delivery of microRNA 21 inhibitor and doxorubicin to cancer cells using core-shell tecto dendrimers formed via supramolecular host-guest assembly. J Mater Chem B. 2020; 8: 2768-74.

4. Xiong ZJ, Alves CS, Wang JH, Li AJ, Liu JY, Shen MW, et al. Zwitterion-functionalized dendrimer-entrapped gold nanoparticles for serum-enhanced gene delivery to inhibit cancer cell metastasis. Acta Biomater. 2019; 99: 320-9.

5. Yong Y, Zhang CF, Gu ZJ, Du JF, Guo Z, Dong XH, et al. Polyoxometalate-Based Radiosensitization Platform for Treating Hypoxic Tumors by Attenuating Radioresistance and Enhancing Radiation Response. ACS Nano. 2017; 11: 7164-76.

6. Fan Y, Tu WZ, Shen MW, Chen XM, Ning YS, Li JJ, et al. Targeted Tumor Hypoxia Dual-Mode CT/MR Imaging and Enhanced Radiation Therapy Using Dendrimer-Based Nanosensitizers. Adv Funct Mater 2020; 30: 1909285.

7. Meng LT, Cheng YL, Tong XN, Gan SJ, Ding YW, Zhang Y, et al. Tumor Oxygenation and Hypoxia Inducible Factor-1 Functional Inhibition via a Reactive Oxygen Species Responsive Nanoplatform for Enhancing Radiation Therapy and Abscopal Effects. ACS Nano. 2018; 12: 8308-22.
